# Supplementary material for: Precision medicine in laryngeal cancer: protocol of the laryngeal cancer cohort (LARCH)
Source: BMJ Open. 2023 Jan 13;13(1):e067561. doi: 10.1136/bmjopen-2022-067561 (PMC9843189; doi:10.1136/bmjopen-2022-067561)
Supplement: Author's manuscript [file bmjopen-2022-067561.draft_revisions.pdf]

BMJ Open

BMJ Open is committed to open peer review. As part of this commitment we make the peer review history of every article we publish publicly available.

When an article is published we post the peer reviewers' comments and the authors' responses online. We also post the versions of the paper that were used during peer review. These are the versions that the peer review comments apply to.

The versions of the paper that follow are the versions that were submitted during the peer review process. They are not the versions of record or the final published versions. They should not be cited or distributed as the published version of this manuscript.

BMJ Open is an open access journal and the full, final, typeset and author-corrected version of record of the manuscript is available on our site with no access controls, subscription charges or pay-per-view fees (http://bmjopen.bmj.com).

If you have any questions on BMJ Open's open peer review process please email info.bmjopen@bmj.com

BMJ Open

Precision medicine in laryngeal cancer: protocol of the laryngeal cancer cohort (LARCH)

Journal:	*BMJ Open*
Manuscript ID	bmjopen-2022-067561
Article Type:	Protocol
Date Submitted by the Author:	07-Sep-2022
Complete List of Authors:	Hamilton, David; Newcastle University, Population Health Sciences Institute; Newcastle Upon Tyne Hospitals NHS Foundation Trust, ENT<br>O'Hara, James; Newcastle Upon Tyne Hospitals NHS Foundation Trust, ENT; Newcastle University, Population Health Sciences Institute<br>Rajgor, Amarkumar; Newcastle Upon Tyne Hospitals NHS Foundation Trust, ENT; Newcastle University, Population Health Sciences Institute<br>Selby, Gerald; Newcastle Upon Tyne Hospitals NHS Foundation Trust, ENT<br>Anderson, Mhairi; Newcastle Upon Tyne Hospitals NHS Foundation Trust, Newcastle Joint Research Office<br>Keltie, Kim; Newcastle Upon Tyne Hospitals NHS Foundation Trust, Northern Medical Physics and Clinical Engineering; Newcastle University, Translational and Clinical Research Institute<br>Parker, Rosalyn; Newcastle Upon Tyne Hospitals NHS Foundation Trust, Northern Medical Physics and Clinical Engineering<br>Teare, Dawn; Newcastle University Faculty of Medical Sciences, Population Health Sciences Institute<br>Patterson, Joanne; University of Liverpool, Liverpool Head and Neck Centre<br>Jones, Terry; Liverpool University, Liverpool Head and Neck Centre<br>Sharp, Linda; Newcastle University, Population Health Sciences Institute
Keywords:	EPIDEMIOLOGY, Head & neck tumours < ONCOLOGY, Head & neck surgery < OTOLARYNGOLOGY, Head & neck imaging < RADIOLOGY & IMAGING, Adult radiotherapy < RADIOTHERAPY, Head & neck surgery < SURGERY

SCHOLARONE™
Manuscripts

Title: Precision medicine in laryngeal cancer: protocol of the laryngeal cancer cohort (LARCH)

Authors

Mr David Hamilton, Consultant Otolaryngology, Head and Neck Surgery, Freeman Hospital Newcastle upon Tyne, Honorary Senior Lecturer, Newcastle University
Address: ENT Department, Newcastle Upon Tyne Hospitals NHS Foundation Trust, Level 3, Freeman Hospital, High Heaton, Newcastle upon Tyne, NE7 7DN
Tel: 0191 2137625
E-mail: David.hamilton@ncl.ac.uk
Role: corresponding author, research question, research design, manuscript preparation, lead on revisions

Mr James O'Hara, Clinical Senior Lecturer, Newcastle University, Consultant Otolaryngology, Head and Neck Surgery, Freeman Hospital Newcastle upon Tyne
Role: research question, research design, manuscript revisions

Mr Amarkumar Rajgor, Academic Clinical Fellow, Otolaryngology, Freeman Hospital Newcastle upon Tyne
Role: research question, research design, manuscript revisions

Mr Gerald Selby, Patient, Freeman Hospital, Newcastle upon Tyne
Role: research design and conduct

Mhairi Anderson, Quality Assurance and Development Manager, Newcastle Joint Research Office.
Role: research governance, quality assurance, manuscript revision

Kim Keltie, Associate Researcher, Translational and Clinical Research Institute, Newcastle University, Newcastle upon Tyne;
Lead Clinical Scientist, Northern Medical Physics and Clinical Engineering, The Newcastle upon Tyne Hospitals NHS Foundation Trust.
Role: research governance and safety, manuscript revisions

Rosalyn Parker, Lecturer in Audiology, Queen Margaret University, Edinburgh
Evaluation Healthcare Scientist, Northern Medical Physics and Clinical Engineering, The Newcastle upon Tyne Hospitals NHS Foundation Trust.
Role: research governance and safety, manuscript revisions

Professor Dawn Teare, Professor of Biostatistics, Population Health Sciences Institute, Newcastle University, England
Role: analysis strategy, manuscript revisions

For peer review only

Prof Joanne Patterson, Professor Speech and Language Therapy, Liverpool Head and Neck Centre, University of Liverpool
Role: research design, outcome measures, manuscript revision

Prof Terry M Jones, Professor of Head and Neck Surgery, Liverpool Head and Neck Centre, University of Liverpool
Role: research question, research design, manuscript revisions

Professor Linda Sharp, Professor of Cancer Epidemiology, Population Health Sciences Institute, Newcastle University, England
Role: research question, research design, manuscript revisions

Funding statement

This work was supported by the MRC and NIHR via the Clinical Academic Research Partnership (CARP) funding stream, grant number MR/V037528/1

Competing interests statement

All authors have no relationships with any companies that might have an interest in the submitted work in the previous 3 years and no non-financial interests that may be relevant to the submitted work.

Word count: 3860

For peer review only

For peer review only - http://bmjopen.bmj.com/site/about/guidelines.xhtml

Abstract

Introduction

Laryngeal cancer disproportionately affects socioeconomically disadvantaged patients. Treatment can render a patient nil by mouth or in need of a permanent tracheostomy. In the past 30 years survival has remained at best static and at worst it has declined. Currently, there is no method of prognosticating how a patient will respond to treatment.

The LARyngeal Cancer coHort (LARCH) aims to establish how survival and quality of life outcomes compare between surgery and (chemo)radiotherapy in early and advanced laryngeal cancer and how the presenting features of laryngeal cancer influence oncological, functional and quality of life outcome.

Methods and analysis

This study is the first enhanced laryngeal cancer disease cohort. In the initial phase, we aim to deliver a prospective cohort study of 150 patients in eight centres over a 3-year period.

Patient, tumour, quality of life and laryngeal functional data will be collected and baseline, 6, 12 and 24 months. Multiple logistic regression analyses will be used to quantify locoregional control and identify factors associated with control overall and by treatment modality and identify factors associated with quality-of-life overall and by treatment modality.

Ethics and dissemination

Most interventions take place as part of routine care, with LARCH providing a mechanism for recording this data centrally. When successfully recruiting in the North of England, we plan to roll-out LARCH nationwide; in the future, LARCH can be used as a trial platform in the disease. The results will be submitted for publication in high-impact international peer-reviewed journals and presented to scientific meetings. Access to the anonymised LARCH dataset by other researchers will be publicised and promoted.

Registration

Sponsor: The Newcastle upon Tyne Hospitals NHS Foundation Trust

Newcastle University: NU-005954

Newcastle upon Tyne Hospitals Research and Development: 09972

Integrated Research Application Service Number: 306921

Central Portfolio Management System (CPMS) ID: 52643

ISRCTN 27819867

For peer review only

For peer review only - http://bmjopen.bmj.com/site/about/guidelines.xhtml

Strength and limitations of this study

- This is a pragmatic observational study, collecting real-world data, for which most patients presenting to participating centres will be eligible.
- The specific data collected will have value in predicting response to treatment measured both in terms of clinical and patient-reported outcomes; this has not been collected before on this scale for laryngeal cancer
- Research processes allow the routine collection of radiological and tissue data, linked to patient-reported and clinical outcomes
- While the effectiveness of treatments are being compared, and rigorous methods will be used to control confounders, some care will be needed in interpretation of findings due to the observational design.

For peer review only

Introduction

Laryngeal cancer affects 2400 new patients each year in the UK. Incidence increases with age and the cancer disproportionately affects those who are socioeconomically disadvantaged[1]. Around half of patients present with advanced disease and, consequently, have poor survival. Those affected are often left with a significantly diminished quality of life: treatment can render the individual permanently nil by mouth due to swallowing problems or in need of a permanent tracheostomy or tracheal stoma. The impact on quality of voice or communication can prevent effective communication. Swallowing difficulty and dry mouth can necessitate permanent gastrostomy tube feeding with effects on socialising and relationships. Laryngeal cancer comprises early (T1 and T2) and advanced (T3 and T4) cancer. Progress in the treatment of both has stalled over the past 30 years. There has been a sea-change in treatment algorithms, with increasing use of transoral laser in early disease and the advent of “laryngeal preservation” in advanced disease. But, over the same time period, survival has remained at best static, and at worst it has declined. From a wider societal perspective, laryngeal cancer diagnosis and treatment costs the NHS £96 million per year with estimated productivity losses of £230,000/patient[2].

The last reported randomised controlled trial (RCT) which successfully recruited patients and compared surgical and nonsurgical treatments reported in 1991[3]; subsequent RCTs have either compared non-surgical treatments alone or failed to recruit successfully[4 5]. We have investigated the possibilities of clinical trials in this field, and have recognised the work done to attempt to select patients on the basis of response to up front (induction) chemotherapy[6]. However, induction chemotherapy has repeatedly shown decreased disease control rates[4]. Also, there is no consensus on how tumour response should be measured or defined in RCTs. Moreover, researchers’ and clinicians’ outcome priorities of locoregional control and organ preservations are not necessarily shared by the patient population; indeed, our group has shown that patients will prioritise swallow and voice function over treatment modality or survival[7]. Currently, clinicians have no method of prognosticating how a specific tumour or patient will respond to treatment. For example, one patient with advanced disease treated with radiotherapy will achieve good locoregional control, preserve their larynx, achieve a functional swallow and produce their voice normally. Another patient with the same stage of disease, treated with the same modality, may have persistent disease after 6 weeks of treatment, or will be rendered nil by mouth, gastrostomy fed and dependent on a breathing tube. This has led to clinicians relying on experience and anecdote to guide and support patients though complex decisions. This, in turn, has led to huge variation in treatments delivered across the UK; for example the rate of primary radiotherapy for advanced laryngeal cancer varies across centres from 0% to 83%[8].

To move forward, we require an understanding of how an individual patient with laryngeal cancer responds to treatment, with respect to both tumour control and functional outcome. Further, clinicians and researchers need to be able to pre-determine this response at presentation. Knowledge of the factors which dictate response to treatment would allow researchers to identify the variables to control for when designing future RCTs of novel treatments. However, risk prediction or precision medicine in laryngeal cancer remains elusive. Clinical cohorts, enriched with rich clinical data and biological samples, are an invaluable resource for advancing precision medicine. In head and neck cancer, audits and previous cohort studies have examined, to some extent, processes of care and survival[9 10]. This has allowed some initial hypothesis generation; however ambitions to include patients with all cancers have limited the usefulness of the data and clinical engagement. The largest such cohort, the

For peer review only

“Head and Neck 5000 study” (HN5000), collected longitudinal data on 5000 patients (1065 with laryngeal cancer) with 3 year survival and patient reported outcomes, but clinical data and samples are limited. However, the endeavour does establish proof of concept and a blueprint for a specific laryngeal cancer cohort.

In other cancers sites (breast, prostate, lymphoma), biochemical analysis of biopsy samples allows the tailoring of specific treatments to an individual tumour; unfortunately in laryngeal cancer researchers have not made such progress. This is partly due to the heterogeneous tumour biology of carcinogen-induced malignancies and partly because this cancer attracts little research interest and spending (0.8% of cancer research spend in 2018/19[11]). There are potential prognostic markers under investigation: either patient-based (tobacco use, muscle mass) or tumour-driven (the immune checkpoint inhibitor complex IDO, ‘immunoscore’, PDL-1). Although the latter are under investigation for response to novel chemotherapeutic agents, their presence does not currently influence the choice of primary treatment[12]. The emerging field of “radiomics” uses the raw data from routine CT scans and maps this to tumour and patient outcomes as a “radiological biomarker”[13]. This means that although every patient with laryngeal cancer undergoes tissue biopsy and cross-sectional imaging, much of the data goes to waste.

The LARyngeal Cancer coHort (LARCH) - a cohort study of all laryngeal cancers, enriched with samples and detailed clinical data - is a major step towards defining precision medicine in laryngeal cancer and, in the longer term, to improving patient outcomes. It will allow significant progress in areas including: comparative (treatment) effectiveness research; investigation of biochemical and radiological biomarkers (mapped to treatment outcome); mapping clinical and patient characteristics to outcome; patient involvement in decision making; and the identification of variables for further investigation in trials of novel prognosticators or therapeutics.

Primary objectives

To establish a disease database of laryngeal cancer patients in order to:

1. Assess the difference in quality of life, disease specific and overall survival between treatment modalities in early and advanced laryngeal cancer.
2. Assess the impact of patient-derived clinical features and tumour factors on treatment outcome (oncological, laryngeal function, quality of life, swallow, voice) in early and advanced laryngeal cancer and use this to develop a risk prediction tool.

Secondary objectives

1. To establish consent processes to allow researchers to re-contact patients for data on long term outcome and survivorship.
2. Using the data, establish an initial risk communication tool in the disease.
3. To develop the pathway for routine tissue and radiological scan collection for future studies, mapped to outcome.

For peer review only - http://bmjopen.bmj.com/site/about/guidelines.xhtml

Methods and analysis

This study is the first enhanced laryngeal cancer disease cohort. We aim to deliver a prospective cohort study of a minimum of 150 patients recruited over a 3-year period. This initial phase of the study will recruit patients from eight head and neck cancer centres in the North of England. Once established, in future research, we aim to roll out the cohort nationally.

This study will be pragmatic, involving patients with laryngeal cancer attending hospital as part of their routine care. Patients will be identified by a member of the research team and recruitment will be supported by the research team.

Eligibility criteria

Inclusion criteria

- Suspected but unconfirmed laryngeal cancer (Group 1)
- Confirmed new diagnosis of laryngeal cancer (Group 2)
- Age over 18
- Capacity to consent
- Ability to understand written and spoken English

Exclusion criteria

- Recurrence or second head and neck primary cancer

Patients from Group 1 who are ineligible for inclusion into Group 2 will be withdrawn from the study and subjects replaced. Patients who withdraw from the study during the enrolment period will be replaced. Withdrawn subjects will be replaced until at least 150 eligible patients have completed the study endpoints.

Group 1: suspected laryngeal cancer

Patients with a suspected diagnosis of laryngeal cancer will be identified by the clinical team at the time that laryngeal cancer is suspected. The patient will be given the participant information sheet (PIS) by the clinical team at the time of the clinic appointment or sent a copy of the PIS by a research nurse after the clinic appointment has taken place. On the day of the biopsy, they will be approached by a member of the research team and given an opportunity to ask any questions about the study. If the biopsy does not confirm cancer, the patient will be withdrawn from the study and any data collected so far will be destroyed. Patients with a biopsy confirming laryngeal cancer will be eligible to continue in the study and move to Group 2.

Group 2: confirmed new laryngeal cancer diagnosis

Patients who have laryngeal cancer confirmed by biopsy who have not been enrolled into the study previously will be identified by clinical staff at the clinic following their diagnosis of laryngeal cancer by biopsy. At the time of diagnosis, they will be given the PIS, consent form and the preliminary questionnaires.

For peer review only

For peer review only - http://bmjopen.bmj.com/site/about/guidelines.xhtml

Consent

Potential participants will be assessed by a member of the study team, eligibility will be confirmed and baseline assessments performed. Consent to enter the study will be sought from each participant only after a full explanation has been given and the PIS has been given. We have worked with our patient and public involvement (PPI) representatives to ensure that these materials are concise, clear and easy to understand.

The patient is free to refuse participation without giving reasons and without this affecting the care they receive. The participant is free to withdraw at any time from the study without giving reasons and without prejudicing their further treatment. Participants are provided with a contact point where they may obtain further information about the study in the PIS. Data and samples collected up to the point of withdrawal will be kept.

Consent to additional elements of the research will be driven by patients' wishes giving them control over how their data is used. Patients will be offered the opportunity to consent to the following without affecting their inclusion within the core aspects of the study:

- Consent for future contact with information about studies that become available
- Consent for future contact for collection of additional information regarding this study
- Use of anonymised information collected from this study to be used in future studies which have received separate ethical approval
- All patients who are identified before their biopsy, have a cancer recurrence or who have a resection will be eligible for consent for storage of their tissue. This consent details that their tissue will be stored indefinitely and can be used for future, as yet undecided studies. Patients have the option to give consent for further biopsies (often in the case of recurrence) to be collected and transferred to a biobank for use in future research projects.

Patients may consent to as many or few additional elements as they wish.

Data collection

The following data will be collected on all patients; additional study questionnaires will be collected at any time before the patient begins treatment for their laryngeal cancer, Table 1.

For peer review only

For peer review only - http://bmjopen.bmj.com/site/about/guidelines.xhtml

Table 1: Data collected at baseline assessment

Clinical	Radiological	Functional	Histology	Treatment received
Age	Laryngeal subsite	EORTC QLQ-C30	From biopsy	Primary non-surgical treatment<br>- Chemotherapy agent and dose<br>- Radiotherapy dose and fractionation
Date of birth	Cartilaginous framework involvement	EORTC HN35	Histology	Primary surgical management<br>- Procedure performed
Gender at birth	Maximum dimension	Vocal cord function	Grade	Post-operative chemotherapy
NHS number	Extralaryngeal spread	DIGEST VFSS/FEES scale	Differentiation	Post-operative radiotherapy
Treatment hospital	Bilateral involvement	PSS-HN	Digital photography if available	Date of completion of treatment
GP Postcode	Tumour max SUV	MDADI	From resection (if performed)	
Date of biopsy	Signs of aspiration	VHI-10	Digital photography if available	
Date of initiation of treatment	Cervical lymph node involvement	100 mLs Water swallow test	Primary site	
Aim of treatment	Evidence of extra-capsular spread	Maximum phonation time	Dimension	
TNM stage		GRBAS scale	Primary site	
Smoking history		Voice recording	Uni/multifocal	
Alcohol history			Histology	
ACE score			Differentiation	
Clinical frailty scale			Invasiveness	
WHO performance			Invasive front	
Weight			Perineural invasion	
Presence of tube feeding			Lymphovascular invasion	
			Closest margin	
			Pathological TNM staging	
			Neck disease	
			Total number of nodes dissected	
			Total number of involved nodes	

For peer review only

Patients will be followed up until 24 months (+/- 3 months) after the completion of their last treatment, or until date of death if this occurs during the study. Frequency of follow-up will follow standard care (approximately 6, 12 and 24 months), with clinical and functional information collected at each follow-up visit, Table 2.

Table 2: Data collected at follow-up assessment

Clinical	Functional
Disease status<br>Alive without disease<br>Alive with disease<br>Death (disease related)<br>Death (non-disease related)	EORTC QLQ-C30
Smoking history	EORTC HN35
Alcohol history	Vocal cord function
ACE score	DIGEST VFSS/FEES scale
Weight	PSS-HN
Presence of tube feeding	MDADI
	VHI-10
	100 mLs Water swallow test
	Minimum phonation time
	GRBAS scale
	Voice recording

If a patient has a scan as part of their routine care, the scan will be stored and the radiological data will be reported and collected as above

If a patient has a cancer recurrence, as well as the radiological data outlined above, the tissue will be collected and stored in a biobank as discussed in the consent section above. In the event of a recurrence, the data collected will be the same as a primary disease presentation.

Storage and analysis of biological samples

Tumour tissue biopsies at diagnosis, during treatment or on relapse will be obtained or at surgical resection at presentation, during treatment or on relapse in line with participant's consent.

The procedure for sample retrieval will depend on the nature of the sample requested and the laboratory services on site. If consent is withdrawn for issued samples by the donor, recipients will be informed of the relevant sample numbers and asked to return any unused samples for destruction. Results obtained from samples that have already been used for research need not be destroyed.

For peer review only

Routine imaging and radiomic analysis

All scans taken as part of routine care will be stored on hospital servers (as is usual practice). If not already in Newcastle, many of these scans will be transferred using the routine PACS transfer service, to Newcastle upon Tyne Hospital NHS Foundation Trust. These scans will be subjected to radiomic analysis: scans will be extracted in DICOM format anonymised ensuring removal of all patient identifiable information.

Statistical Analysis

Patient data and information will be collected, stored and used based upon patient consent, and consistent with GDPR requirements. Descriptive statistics will be used to describe the recruited cohort and data completion (at baseline and at follow-up). Baseline characteristics will be compared with routine data (from Hospital Episode Statistics) to determine whether the recruited sample is representative of laryngeal cancer patients across NHS in England.

Depending on the maturity of the cohort at the conclusion of the study, data will be used to start to develop risk prediction models and compare outcomes by treatment modality. Multiple logistic regression analyses will be used to (i) identify patient-related, clinical and health service-related (e.g. institution) factors associated with receipt of surgical vs non-surgical treatment; (ii) quantify locoregional control and identify factors associated with control overall and by treatment modality; and (iii) identify factors associated with quality-of-life overall and by treatment modality. Epidemiological approaches to support treatment comparisons within observational datasets (e.g. propensity scores, instrumental variable analysis) will be used. These analyses will provide an early demonstration of the value and potential of this new cohort.

The planned analyses will draw on a range of epidemiological analytical approaches. They will explore utility of high dimensional propensity scores (which incorporate additional variables in the propensity score, such as clinician and/or hospital characteristics, with these serving as proxies for unmeasured confounders) and instrumental variable analysis (which relies on the existence of an ‘instrument’, a variable that is related to the treatment but not to the study outcome other than through treatment effects).

Ethics and Dissemination

The study will be conducted in accordance with the recommendations for physicians involved in research on human subjects adopted by the 18th World Medical Assembly, Helsinki 1964 and later revisions. The majority of the interventions delivered take place as part of routine care, with LARCH providing a mechanism for recording this data. Biological material will be stored under each establishment Human Tissue Authority (HTA) licence. Ethical approval for the project has been obtained from London - Surrey Borders Research Ethics Committee, reference 22/PR/0406. The project has been included in the NIHR portfolio, CPMS ID: 52643. All potential future studies will have separate ethical approval.

Confidentiality and data management

The Caldicott Principles and General Data Protection Regulation (GDPR) will be fully adhered to when dealing with patient identifiable data. Within each recruiting site the Principal Investigator will preserve the confidentiality of participants taking part in the study. Within sites, no staff beyond the usual care team and local research team will have access to identifiable data. The data will be held at the site in

For peer review only - http://bmjopen.bmj.com/site/about/guidelines.xhtml

accordance with local Trust policies and will be destroyed following the study close in accordance with local Research and Development protocols.

The data controller (Sponsor) will have access to the full dataset but other users will be restricted to data from their own centre. Access will be via secure portal. Local sites will have access to their site data.

A purpose-built database and web portal will be developed to support the capture of patient recruitment and study participation data. Only authorised individuals will have login credentials to input this data. The staff (including research nurses within recruiting centres) will be able to remote access the portal to transfer the data. Staff will be a delegated out by Principal Investigator based on skills, training and experience; these will be used to restrict access to confidential or otherwise sensitive data. Our web portal will be deployed using REDCap is a browser-based Electronic Case Report Form (eCRF), suitable for Newcastle-led non-commercial research studies. This system site is on HSCN (i.e. the data is securely behind the NHS firewall).

Data generated by the research project will be retained after publications resulting from the research are finalised. Data will be stored for 20 years. After 20 years data will be considered for longer-term retention based on the published results and further advances in the field of research internationally. Datasets stored for >20 years will be anonymised.

At the end of the study, strategies to promote and publicise the cohort and processes for gaining access to data, samples or the recruitment network will be developed and widely publicized and promoted. These will include: information on the cohort website; designated contact point for further information or support; flyers, stalls or sessions at scientific and stakeholder conferences; webinars or podcasts, and providing a DOI created through data.ncl for inclusion on the project website and published papers.

The data sharing policy, and the procedures (including application forms) for requesting access to the cohort, will be made available on the cohort website. Currently it is anticipated that applications will be prioritised based on: quality; alignment with our vision; potential to translate into clinical practice or deliver significant patient or public benefit; collaborative approach; and added value. The cohort research committee will have responsibility for reviewing and approving/declining requests for access.

Data held in the cohort database will be offered for sharing once: (i) the principles on which applications will be reviewed, prioritised and approved are agreed within the collaboration; (ii) the processes for applying to access the cohort are agreed and implemented; (iii) reasonable numbers of patients have been recruited to the cohort. Investigators of adopted research studies will develop their own timelines for data sharing. A requirement of cohort approval will be that data are made available for sharing at as early a point as possible. For these studies, we currently anticipate that the applicant, research partner and other partners and collaborators will have access to the data for 1-2 years after the end of data collection for the study. Patients recruited to the cohort will be asked for consent to data sharing. For the cohort-adopted studies, it will be a requirement of adoption that consent is obtained from recruits for data sharing. The major delay to data sharing which will occur relates to the need to establish procedures for study review and adoption

The SMG will make the decision on data access. Committee membership will include the chief investigator (DWH) and co-investigators (JOH and LS) and PPI representation. This group will act as gatekeepers for the data. Decisions on whether or not an application is approved will be recorded

For peer review only - http://bmjopen.bmj.com/site/about/guidelines.xhtml

together with, in the event the application is declined, reasons and whether the applicants may re-apply if they fulfil certain conditions.

The Newcastle upon Tyne Hospitals NHS Foundation Trust, as data controller, will require data sharing agreements with all organisations responsible for submitting and accessing data.

Patients will have their date of birth and NHS number collected and this will be stored in a secure purpose built database. Where data linkage to national databases such as NHS Digital, National Cancer registries or Hospital Episode statistics is required, this identifiable information will be passed onto these national organisation to allow data linkage.

Patients who have withdrawn consent will have all data collected up until the point of withdrawal included in the study. This data will be uploaded onto an electronic CRF and included in the analysis of the study. Data will be submitted either directly onto the electronic CRF or onto paper CRF before input into the electronic CRF. In relation to consent for contact, researchers undertaking studies requiring access to identifiable data (e.g.in order to contact patients about participation in a new research study) will be granted appropriate access on the basis of agreement from the study management team and subject to ethical approval and consistent with patient consent.

Use of tumour samples or data by other researchers

Researchers may request to use material from the LARCH study which have been stored in Biobanks for future research. Researchers will be required to have their own NHS research ethics committee approval for their research projects, and the release of the material must be approved by the LARCH access committee to ensure an appropriate use of samples. This will be conducted in accordance with a formalised Access Policy and procedure. Access to the tissue collection is available to research groups based in the UK and elsewhere.

Patient and Public Involvement

We explored multiple potential study designs during the preparation of this protocol with three patient and public involvement (PPI) groups; these groups were not in favour of a randomised trial design. In defining precision medicine in laryngeal cancer, we do not aim to show whether a particular treatment is “better” for patients. Rather we aim to specify the tumour and patient factors which lead to a poor response to laryngeal cancer treatment (in general, and for specific treatments) in individual patients. PPI groups have checked and approved all patient facing study documents. We have a patient representative on the study management group and a patient (GS) is a co-author of this protocol and was involved in manuscript preparation

Sponsor

LARCH will be led by Newcastle University with The Newcastle upon Tyne Hospitals NHS Foundation Trust acting as sponsor. In participating sites, a principal investigator will be identified. Portfolio adoption will ensure that NIHR CRN research nurses are able to undertake patient recruitment.

Study management

LARCH will be coordinated by a study management group (SMG). The SMG will be chaired by the chief investigator and comprise co-investigators, PPI representation, study manager, research fellow, lead nurse, data managers and digital research engineers. The SMG will advise on matters including appropriateness and sensitivity of patient-facing materials; methods of patient approach, consent and data collection; strategies to maximise retention; interpretation of results; and dissemination to lay

For peer review only - http://bmjopen.bmj.com/site/about/guidelines.xhtml

audiences. Active involvement of the will help maximise patient acceptability and, hence, recruitment and retention. The study management team will govern all access to data.

A representative group of staff who will be part of the research team at each site will attend a site initiation meeting to ensure compliance with the protocol and allow training in study procedures and data collection methods. The PI at each study site will apply for local R&D approval. The PI will sign a copy of the ethically approved protocol to confirm agreement to carry out all study related tasks in accordance with the protocol. Deviations from protocol will be reported to the study management group.

Funding

Funding to this study is from The MRC/NIHR Clinical Academic Research Partnership (CARP), and is on NIHR portfolio (CPMS: 52643) gaining additional support in identifying patients and gaining consent.

Dissemination

Data from the study will be disseminated to participating centres within the study via the Trust's websites. Additionally, lay summaries will be prepared, posted on the study website and disseminated through websites of individual participating sites, charities, and locations advised by PPI. Digital tools for dissemination of research findings will be developed and information disseminated to individuals requesting this.

For academic and clinical dissemination, the results will be submitted for publication in high-impact international peer-reviewed journals and presented to scientific meetings.

For peer review only

For peer review only - http://bmjopen.bmj.com/site/about/guidelines.xhtml

References

1. CRUK. Head and Neck Cancer Incidence Statistics 2015 [Available from: https://www.cancerresearchuk.org/health-professional/cancer-statistics/statistics-by-cancer-type/head-and-neck-cancers/incidence#heading-Four accessed 20/05/2019 2019.
2. Pearce AM, Hanly P, Timmons A, et al. Productivity Losses Associated with Head and Neck Cancer Using the Human Capital and Friction Cost Approaches. *Appl Health Econ Health Policy* 2015;13(4):359-67. doi: 10.1007/s40258-015-0155-8 [published Online First: 2015/02/19]
3. Induction chemotherapy plus radiation compared with surgery plus radiation in patients with advanced laryngeal cancer. The Department of Veterans Affairs Laryngeal Cancer Study Group. *The New England journal of medicine* 1991;324(24):1685-90. doi: 10.1056/NEJM199106133242402
4. Forastiere AA, Goepfert H, Maor M, et al. Concurrent chemotherapy and radiotherapy for organ preservation in advanced laryngeal cancer. *The New England journal of medicine* 2003;349(22):2091-8. doi: 10.1056/NEJMoa031317
5. Hamilton DW, de Salis I, Donovan JL, et al. The recruitment of patients to trials in head and neck cancer: a qualitative study of the EaStER trial of treatments for early laryngeal cancer. *European archives of oto-rhino-laryngology : official journal of the European Federation of Oto-Rhino-Laryngological Societies* 2013 doi: 10.1007/s00405-013-2349-8
6. Urba S, Wolf G, Eisbruch A, et al. Single-cycle induction chemotherapy selects patients with advanced laryngeal cancer for combined chemoradiation: a new treatment paradigm. *Journal of clinical oncology : official journal of the American Society of Clinical Oncology* 2006;24(4):593-8. doi: 10.1200/JCO.2005.01.2047
7. Hamilton DW, Bins JE, McMeekin P, et al. Quantity vs quality of life in laryngeal cancer: a time trade off study. *Clinical Otolarygology* 2012;37(Suppl. 1):108.
8. DAHNO. National Head and Neck Cancer Audit 2014, DAHNO Tenth Annual Report 2014 [Available from: https://digital.nhs.uk/data-and-information/publications/statistical/national-head-and-neck-cancer-audit/national-head-and-neck-cancer-audit-2014-dahno-tenth-annual-report2019.
9. Ingarfield K, McMahon AD, Douglas CM, et al. Determinants of long-term survival in a population-based cohort study of patients with head and neck cancer from Scotland. *Head & neck* 2019 doi: 10.1002/hed.25630 [published Online First: 2019/01/09]
10. Birchall M, Bailey D, King P, et al. Effect of process standards on survival of patients with head and neck cancer in the south and west of England. *British journal of cancer* 2004;91(8):1477-81. doi: 10.1038/sj.bjc.6602118
11. NCRI. [Available from: https://www.ncri.org.uk/ncri-cancer-research-database-old/spend-by-research-category-and-disease-site/ accessed Oct 2020.
12. Economopoulou P, de Bree R, Kotsantis I, et al. Diagnostic Tumor Markers in Head and Neck Squamous Cell Carcinoma (HNSCC) in the Clinical Setting. *Front Oncol* 2019;9:827. doi: 10.3389/fonc.2019.00827 [published Online First: 2019/09/27]
13. Rajgor AD, Patel S, McCulloch D, et al. The application of radiomics in laryngeal cancer. *Br J Radiol* 2021;94(1128):20210499. doi: 10.1259/bjr.20210499 [published Online First: 2021/09/30]

For peer review only

STROBE Statement—checklist of items that should be included in reports of observational studies

	Item No	Recommendation	Page No
**Title and abstract**	1	(*a*) Indicate the study's design with a commonly used term in the title or the abstract	1,3
		(*b*) Provide in the abstract an informative and balanced summary of what was done and what was found	3
**Introduction**			
Background/rationale	2	Explain the scientific background and rationale for the investigation being reported	5,6
Objectives	3	State specific objectives, including any prespecified hypotheses	6
**Methods**			
Study design	4	Present key elements of study design early in the paper	7
Setting	5	Describe the setting, locations, and relevant dates, including periods of recruitment, exposure, follow-up, and data collection	9,10
Participants	6	(*a*) *Cohort study*—Give the eligibility criteria, and the sources and methods of selection of participants. Describe methods of follow-up<br>*Case-control study*—Give the eligibility criteria, and the sources and methods of case ascertainment and control selection. Give the rationale for the choice of cases and controls<br>*Cross-sectional study*—Give the eligibility criteria, and the sources and methods of selection of participants	7
		(*b*) *Cohort study*—For matched studies, give matching criteria and number of exposed and unexposed<br>*Case-control study*—For matched studies, give matching criteria and the number of controls per case	
Variables	7	Clearly define all outcomes, exposures, predictors, potential confounders, and effect modifiers. Give diagnostic criteria, if applicable	9,10
Data sources/ measurement	8*	For each variable of interest, give sources of data and details of methods of assessment (measurement). Describe comparability of assessment methods if there is more than one group	*9,10*
Bias	9	Describe any efforts to address potential sources of bias	11
Study size	10	Explain how the study size was arrived at	7
Quantitative variables	11	Explain how quantitative variables were handled in the analyses. If applicable, describe which groupings were chosen and why	11
Statistical methods	12	(*a*) Describe all statistical methods, including those used to control for confounding	11
		(*b*) Describe any methods used to examine subgroups and interactions	11
		(*c*) Explain how missing data were addressed	7,8
		(*d*) *Cohort study*—If applicable, explain how loss to follow-up was addressed<br>*Case-control study*—If applicable, explain how matching of cases and controls was addressed<br>*Cross-sectional study*—If applicable, describe analytical methods taking account of sampling strategy	7,8
		(*e*) Describe any sensitivity analyses	

Continued on next page

For peer review only

**Results**			
Participants	13*	(a) Report numbers of individuals at each stage of study—eg numbers potentially eligible, examined for eligibility, confirmed eligible, included in the study, completing follow-up, and analysed	N/A
		(b) Give reasons for non-participation at each stage	N/A
		(c) Consider use of a flow diagram	N/A
Descriptive data	14*	(a) Give characteristics of study participants (eg demographic, clinical, social) and information on exposures and potential confounders	N/A
		(b) Indicate number of participants with missing data for each variable of interest	N/A
		(c) *Cohort study*—Summarise follow-up time (eg, average and total amount)	N/A
Outcome data	15*	*Cohort study*—Report numbers of outcome events or summary measures over time	N/A
		Case-control study—Report numbers in each exposure category, or summary measures of exposure	N/A
		Cross-sectional study—Report numbers of outcome events or summary measures	N/A
Main results	16	(*a*) Give unadjusted estimates and, if applicable, confounder-adjusted estimates and their precision (eg, 95% confidence interval). Make clear which confounders were adjusted for and why they were included	N/A
		(*b*) Report category boundaries when continuous variables were categorized	N/A
		(*c*) If relevant, consider translating estimates of relative risk into absolute risk for a meaningful time period	N/A
Other analyses	17	Report other analyses done—eg analyses of subgroups and interactions, and sensitivity analyses	N/A
**Discussion**			
Key results	18	Summarise key results with reference to study objectives	N/A
Limitations	19	Discuss limitations of the study, taking into account sources of potential bias or imprecision. Discuss both direction and magnitude of any potential bias	N/A
Interpretation	20	Give a cautious overall interpretation of results considering objectives, limitations, multiplicity of analyses, results from similar studies, and other relevant evidence	N/A
Generalisability	21	Discuss the generalisability (external validity) of the study results	N/A
**Other information**			
Funding	22	Give the source of funding and the role of the funders for the present study and, if applicable, for the original study on which the present article is based	14

*Give information separately for cases and controls in case-control studies and, if applicable, for exposed and unexposed groups in cohort and cross-sectional studies.

Note: An Explanation and Elaboration article discusses each checklist item and gives methodological background and published examples of transparent reporting. The STROBE checklist is best used in conjunction with this article (freely available on the Web sites of PLoS Medicine at http://www.plosmedicine.org/, Annals of Internal Medicine at http://www.annals.org/, and Epidemiology at http://www.epidem.com/). Information on the STROBE Initiative is available at www.strobe-statement.org.

For peer review only

For peer review only - http://bmjopen.bmj.com/site/about/guidelines.xhtml

BMJ Open

Precision medicine in laryngeal cancer: protocol of the laryngeal cancer cohort (LARCH)

Journal:	*BMJ Open*
Manuscript ID	bmjopen-2022-067561.R1
Article Type:	Protocol
Date Submitted by the Author:	21-Dec-2022
Complete List of Authors:	Hamilton, David; Newcastle University, Population Health Sciences Institute; Newcastle Upon Tyne Hospitals NHS Foundation Trust, ENT<br>O'Hara, James; Newcastle Upon Tyne Hospitals NHS Foundation Trust, ENT; Newcastle University, Population Health Sciences Institute<br>Rajgor, Amarkumar; Newcastle Upon Tyne Hospitals NHS Foundation Trust, ENT; Newcastle University, Population Health Sciences Institute<br>Selby, Gerald; Newcastle Upon Tyne Hospitals NHS Foundation Trust, ENT<br>Anderson, Mhairi; Newcastle Upon Tyne Hospitals NHS Foundation Trust, Newcastle Joint Research Office<br>Keltie, Kim; Newcastle Upon Tyne Hospitals NHS Foundation Trust, Northern Medical Physics and Clinical Engineering; Newcastle University, Translational and Clinical Research Institute<br>Parker, Rosalyn; Newcastle Upon Tyne Hospitals NHS Foundation Trust, Northern Medical Physics and Clinical Engineering<br>Teare, Dawn; Newcastle University Faculty of Medical Sciences, Population Health Sciences Institute<br>Patterson, Joanne; University of Liverpool, Liverpool Head and Neck Centre<br>Jones, Terry; Liverpool University, Liverpool Head and Neck Centre<br>Sharp, Linda; Newcastle University, Population Health Sciences Institute
**Primary Subject Heading**:	Ear, nose and throat/otolaryngology
Secondary Subject Heading:	Ear, nose and throat/otolaryngology, Oncology, Health informatics, Health services research
Keywords:	EPIDEMIOLOGY, Head & neck tumours < ONCOLOGY, Head & neck surgery < OTOLARYNGOLOGY, Head & neck imaging < RADIOLOGY & IMAGING, Adult radiotherapy < RADIOTHERAPY, Head & neck surgery < SURGERY

SCHOLARONE™
Manuscripts

Title: Precision medicine in laryngeal cancer: protocol of the laryngeal cancer cohort (LARCH)

Authors

Mr David Hamilton, Consultant Otolaryngology, Head and Neck Surgery, Freeman Hospital Newcastle upon Tyne, Honorary Senior Lecturer, Newcastle University
Address: ENT Department, Newcastle Upon Tyne Hospitals NHS Foundation Trust, Level 3, Freeman Hospital, High Heaton, Newcastle upon Tyne, NE7 7DN
Tel: 0191 2137625
E-mail: David.hamilton@ncl.ac.uk
Role: corresponding author, research question, research design, manuscript preparation, lead on revisions

Mr James O'Hara, Clinical Senior Lecturer, Newcastle University, Consultant Otolaryngology, Head and Neck Surgery, Freeman Hospital Newcastle upon Tyne
Role: research question, research design, manuscript revisions

Mr Amarkumar Rajgor, Academic Clinical Fellow, Otolaryngology, Freeman Hospital Newcastle upon Tyne
Role: research question, research design, manuscript revisions

Mr Gerald Selby, Patient, Freeman Hospital, Newcastle upon Tyne
Role: research design and conduct

Mhairi Anderson, Quality Assurance and Development Manager, Newcastle Joint Research Office.
Role: research governance, quality assurance, manuscript revision

Kim Keltie, Associate Researcher, Translational and Clinical Research Institute, Newcastle University, Newcastle upon Tyne;
Lead Clinical Scientist, Northern Medical Physics and Clinical Engineering, The Newcastle upon Tyne Hospitals NHS Foundation Trust.
Role: research governance and safety, manuscript revisions

Rosalyn Parker, Lecturer in Audiology, Queen Margaret University, Edinburgh
Evaluation Healthcare Scientist, Northern Medical Physics and Clinical Engineering, The Newcastle upon Tyne Hospitals NHS Foundation Trust.
Role: research governance and safety, manuscript revisions

Professor Dawn Teare, Professor of Biostatistics, Population Health Sciences Institute, Newcastle University, England
Role: analysis strategy, manuscript revisions

For peer review only - http://bmjopen.bmj.com/site/about/guidelines.xhtml

Prof Joanne Patterson, Professor Speech and Language Therapy, Liverpool Head and Neck Centre, University of Liverpool
Role: research design, outcome measures, manuscript revision

Prof Terry M Jones, Professor of Head and Neck Surgery, Liverpool Head and Neck Centre, University of Liverpool
Role: research question, research design, manuscript revisions

Professor Linda Sharp, Professor of Cancer Epidemiology, Population Health Sciences Institute, Newcastle University, England
Role: research question, research design, manuscript revisions

Word count: 3860

For peer review only

For peer review only - http://bmjopen.bmj.com/site/about/guidelines.xhtml

Abstract

Introduction

Laryngeal cancer disproportionately affects socioeconomically disadvantaged patients. Treatment can render a patient nil by mouth or in need of a permanent tracheostomy. In the past 30 years survival has remained at best static and at worst it has declined. Currently, there is no method of prognosticating how a patient will respond to treatment.

The LARyngeal Cancer coHort (LARCH) aims to establish how survival and quality of life outcomes compare between surgery and (chemo)radiotherapy in early and advanced laryngeal cancer and how the presenting features of laryngeal cancer influence oncological, functional and quality of life outcome.

Methods and analysis

This study is the first enhanced laryngeal cancer disease cohort. In the initial phase, we aim to deliver a prospective cohort study of 150 patients in eight centres over a 3-year period.

Patient, tumour, quality of life and laryngeal functional data will be collected from patients with squamous cell carcinoma of the larynx at baseline, 6, 12 and 24 months. Multiple logistic regression analyses will be used to quantify locoregional control and identify factors associated with control overall and by treatment modality and identify factors associated with quality-of-life overall and by treatment modality.

Ethics and dissemination

Most interventions take place as part of routine care, with LARCH providing a mechanism for recording this data centrally. When successfully recruiting in the North of England, we plan to roll-out LARCH nationwide; in the future, LARCH can be used as a trial platform in the disease. The results will be submitted for publication in high-impact international peer-reviewed journals and presented to scientific meetings. Access to the anonymised LARCH dataset by other researchers will be publicised and promoted.

Registration

Sponsor: The Newcastle upon Tyne Hospitals NHS Foundation Trust

Newcastle University: NU-005954

Newcastle upon Tyne Hospitals Research and Development: 09972

Integrated Research Application Service Number: 306921

Central Portfolio Management System (CPMS) ID: 52643

ISRCTN 27819867

For peer review only - http://bmjopen.bmj.com/site/about/guidelines.xhtml

Strength and limitations of this study

- This is a pragmatic observational study, collecting real-world data, for which most patients presenting to participating centres will be eligible.
- The specific data collected will have value in predicting response to treatment measured both in terms of clinical and patient-reported outcomes; this has not been collected before on this scale for laryngeal cancer
- Research processes allow the routine collection of radiological and tissue data, linked to patient-reported and clinical outcomes
- While the effectiveness of treatments are being compared, and rigorous epidemiological methods will be used to control confounders (such as propensity score matching), some care will be needed in interpretation of findings due to the observational design.

For peer review only

For peer review only - http://bmjopen.bmj.com/site/about/guidelines.xhtml

Introduction

Laryngeal cancer affects 2400 new patients each year in the UK. Incidence increases with age and the cancer disproportionately affects those who are socioeconomically disadvantaged[1]. Around half of patients present with advanced disease and, consequently, have poor survival. Those affected are often left with a significantly diminished quality of life: treatment can render the individual permanently nil by mouth due to swallowing problems or in need of a permanent tracheostomy or tracheal stoma. The impact on quality of voice or communication can prevent effective communication. Swallowing difficulty and dry mouth can necessitate permanent gastrostomy tube feeding with effects on socialising and relationships. Laryngeal cancer comprises early (T1 and T2) and advanced (T3 and T4) cancer. Progress in the treatment of both has stalled over the past 30 years. There has been a sea-change in treatment algorithms, with increasing use of transoral laser in early disease and the advent of "laryngeal preservation" in advanced disease. But, over the same time period, survival has remained at best static[2 3], and at worst it has declined [4]. From a wider societal perspective, laryngeal cancer diagnosis and treatment costs the NHS £96 million per year with estimated productivity losses of £230,000/patient[5].

The last reported randomised controlled trial (RCT) which successfully recruited patients and compared surgical and nonsurgical treatments reported in 1991[6]; subsequent RCTs have either compared non-surgical treatments alone or failed to recruit successfully[7 8]. We have investigated the possibilities of clinical trials in this field, and have recognised the work done to attempt to select patients on the basis of response to up front (induction) chemotherapy[9]. However, induction chemotherapy has repeatedly shown decreased disease control rates[7]. Also, there is no consensus on how tumour response should be measured or defined in RCTs. Moreover, researchers' and clinicians' outcome priorities of locoregional control and organ preservations are not necessarily shared by the patient population; indeed, our group has shown that patients will prioritise swallow and voice function over treatment modality or survival[10]. Currently, clinicians have no method of prognosticating how a specific tumour or patient will respond to treatment. For example, one patient with advanced disease treated with radiotherapy will achieve good locoregional control, preserve their larynx, achieve a functional swallow and produce their voice normally. Another patient with the same stage of disease, treated with the same modality, may have persistent disease after 6 weeks of treatment, or will be rendered nil by mouth, gastrostomy fed and dependent on a breathing tube. This has led to clinicians relying on experience and anecdote to guide and support patients though complex decisions. This, in turn, has led to huge variation in treatments delivered across the UK; for example the rate of primary radiotherapy for advanced laryngeal cancer varies across centres from 0% to 83%[11].

To move forward, we require an understanding of how an individual patient with laryngeal cancer responds to treatment, with respect to both tumour control and functional outcome. Further, clinicians and researchers need to be able to pre-determine this response at presentation. Knowledge of the factors which dictate response to treatment would allow researchers to identify the variables to control for when designing future RCTs of novel treatments. However, risk prediction or precision medicine in laryngeal cancer remains elusive. Clinical cohorts, enriched with rich clinical data and biological samples, are an invaluable resource for advancing precision medicine. In head and neck cancer, audits and previous cohort studies have examined, to some extent, processes of care and survival[12 13].

For peer review only - http://bmjopen.bmj.com/site/about/guidelines.xhtml

This has allowed some initial hypothesis generation; however ambitions to include patients with all cancers have limited the usefulness of the data and clinical engagement. The largest such cohort, the "Head and Neck 5000 study" (HN5000), collected longitudinal data on 5000 patients (1065 with laryngeal cancer) with 3 year survival and patient reported outcomes, but clinical data and samples are limited. However, the endeavour does establish proof of concept and a blueprint for a specific laryngeal cancer cohort.

In other cancers sites (breast, prostate, lymphoma), biochemical analysis of biopsy samples allows the tailoring of specific treatments to an individual tumour; unfortunately in laryngeal cancer researchers have not made such progress. This is partly due to the heterogeneous tumour biology of carcinogen-induced malignancies and partly because this cancer attracts little research interest and spending (0.8% of cancer research spend in 2018/19[14]). There are potential prognostic markers under investigation: either patient-based (tobacco use, muscle mass) or tumour-driven (the immune checkpoint inhibitor complex IDO, 'immunoscore', PDL-1). Although the latter are under investigation for response to novel chemotherapeutic agents, their presence does not currently influence the choice of primary treatment[15]. The emerging field of "radiomics" uses the raw data from routine CT scans and maps this to tumour and patient outcomes as a "radiological biomarker"[16]. This means that although every patient with laryngeal cancer undergoes tissue biopsy and cross-sectional imaging, much of the data goes to waste.

The LARyngeal Cancer coHort (LARCH) - a cohort study of all laryngeal cancers, enriched with samples and detailed clinical data - is a major step towards defining precision medicine in laryngeal cancer and, in the longer term, to improving patient outcomes. It will allow significant progress in areas including: comparative (treatment) effectiveness research; investigation of biochemical and radiological biomarkers (mapped to treatment outcome); mapping clinical and patient characteristics to outcome; patient involvement in decision making; and the identification of variables for further investigation in trials of novel prognosticators or therapeutics.

Primary objectives

To establish a disease database of laryngeal cancer patients in order to:

1. Assess the difference in quality of life, disease specific and overall survival between treatment modalities in early and advanced laryngeal cancer.
2. Assess the impact of patient-derived clinical features and tumour factors on treatment outcome (oncological, laryngeal function, quality of life, swallow, voice) in early and advanced laryngeal cancer and use this to develop a risk prediction tool.

Secondary objectives

1. To establish consent processes to allow researchers to re-contact patients for data on long term outcome and survivorship.
2. Using the data, establish an initial risk communication tool in the disease.
3. To develop the pathway for routine tissue and radiological scan collection for future studies, mapped to outcome.

For peer review only - http://bmjopen.bmj.com/site/about/guidelines.xhtml

Methods and analysis

This study is the first enhanced laryngeal cancer disease cohort. We aim to deliver a prospective cohort study of a minimum of 150 patients recruited over a 3-year period. This initial phase of the study will recruit patients from eight head and neck cancer centres in the North of England: Newcastle, Liverpool, Sunderland, Middlesbrough, Hull, Sheffield, Leeds and Manchester. Once established, in future research, we aim to roll out the cohort nationally.

This study will be pragmatic, involving patients with laryngeal cancer attending hospital as part of their routine care. Patients will be identified by a member of the research team and recruitment will be supported by the research team, all of whom will be trained in Good Clinical Practice and consent.

Eligibility criteria

Inclusion criteria

- Suspected but unconfirmed laryngeal cancer (Group 1)
- Confirmed new diagnosis of laryngeal cancer (Group 2)
- Age over 18
- Capacity to consent
- Ability to understand written and spoken English

Exclusion criteria

- Recurrence or second head and neck primary cancer

Patients from Group 1 who are ineligible for inclusion into Group 2 will be withdrawn from the study and subjects replaced. Patients who withdraw from the study during the enrolment period will be replaced. Withdrawn subjects will be replaced until at least 150 eligible patients have completed the study endpoints.

Group 1: suspected laryngeal cancer

Patients with a suspected diagnosis of laryngeal cancer will be identified by the clinical team at the time that laryngeal cancer is suspected. The patient will be given the participant information sheet (PIS) by the clinical team at the time of the clinic appointment or sent a copy of the PIS by a research nurse after the clinic appointment has taken place. On the day of the biopsy, they will be approached by a member of the research team and given an opportunity to ask any questions about the study. If the biopsy does not confirm cancer, the patient will be withdrawn from the study and any data collected so far will be destroyed. Patients with a biopsy confirming laryngeal cancer will be eligible to continue in the study and move to Group 2.

Group 2: confirmed new laryngeal cancer diagnosis

Patients who have laryngeal cancer confirmed by biopsy who have not been enrolled into the study previously will be identified by clinical staff at the clinic following their diagnosis of laryngeal cancer by biopsy. At the time of diagnosis, they will be given the PIS, consent form and the preliminary questionnaires.

For peer review only

For peer review only - http://bmjopen.bmj.com/site/about/guidelines.xhtml

Consent

Potential participants will be assessed by a member of the study team, eligibility will be confirmed and baseline assessments performed. Consent to enter the study will be sought from each participant only after a full explanation has been given and the PIS has been given. We have worked with our patient and public involvement (PPI) representatives to ensure that these materials are concise, clear and easy to understand.

The patient is free to refuse participation without giving reasons and without this affecting the care they receive. The participant is free to withdraw at any time from the study without giving reasons and without prejudicing their further treatment. Participants are provided with a contact point where they may obtain further information about the study in the PIS. Data and samples collected up to the point of withdrawal will be kept.

Consent to additional elements of the research will be driven by patients' wishes giving them control over how their data is used. Patients will be offered the opportunity to consent to the following without affecting their inclusion within the core aspects of the study:

- Consent for future contact with information about studies that become available
- Consent for future contact for collection of additional information regarding this study
- Use of anonymised information collected from this study to be used in future studies which have received separate ethical approval
- All patients who are identified before their biopsy, have a cancer recurrence or who have a resection will be eligible for consent for storage of their tissue. This consent details that their tissue will be stored indefinitely and can be used for future, as yet undecided studies. Patients have the option to give consent for further biopsies (often in the case of recurrence) to be collected and transferred to a biobank for use in future research projects.

Patients may consent to as many or few additional elements as they wish.

Data collection

The following data will be collected on all patients; additional study questionnaires will be collected at any time before the patient begins treatment for their laryngeal cancer, Table 1.

For peer review only

Table 1: Data collected at baseline assessment

Clinical	Radiological	Functional	Histology	Treatment received
Age	Laryngeal subsite	EORTC QLQ-C30	From biopsy	Primary non-surgical treatment<br>- Chemotherapy agent and dose<br>- Radiotherapy dose and fractionation
Date of birth	Cartilaginous framework involvement	EORTC HN35	Histology	Primary surgical management<br>- Procedure performed
Gender at birth	Maximum dimension	Vocal cord function	Grade	Post-operative chemotherapy
NHS number	Extralaryngeal spread	DIGEST VFSS/FEES scale	Differentiation	Post-operative radiotherapy
Treatment hospital	Bilateral involvement	PSS-HN	Digital photography if available	Date of completion of treatment
GP Postcode	Tumour max SUV	MDADI	From resection (if performed)	
Date of biopsy	Signs of aspiration	VHI-10	Digital photography if available	
Date of initiation of treatment	Cervical lymph node involvement	100 mLs Water swallow test	Primary site	
Aim of treatment	Evidence of extra-capsular spread	Maximum phonation time	Dimension	
TNM stage		GRBAS scale	Primary site	
Smoking history		Voice recording	Uni/multifocal	
Alcohol history			Histology	
ACE score			Differentiation	
Clinical frailty scale			Invasiveness	
WHO performance			Invasive front	
Weight			Perineural invasion	
Presence of tube feeding			Lymphovascular invasion	
			Closest margin	
			Pathological TNM staging	
			Neck disease	
			Total number of nodes dissected	
			Total number of involved nodes	

For peer review only

Patients will be followed up until 24 months (+/- 3 months) after the completion of their last treatment, or until date of death if this occurs during the study. Frequency of follow-up will follow standard care (approximately 6, 12 and 24 months), with clinical and functional information collected at each follow-up visit, Table 2.

Table 2: Data collected at follow-up assessment

Clinical	Functional
Disease status<br>Alive without disease<br>Alive with disease<br>Death (disease related)<br>Death (non-disease related)	EORTC QLQ-C30
Smoking history	EORTC HN35
Alcohol history	Vocal cord function
ACE score	DIGEST VFSS/FEES scale
Weight	PSS-HN
Presence of tube feeding	MDADI
	VHI-10
	100 mLs Water swallow test
	Minimum phonation time
	GRBAS scale
	Voice recording

If a patient has a scan as part of their routine care, the scan will be stored and the radiological data will be reported and collected as above

If a patient has a cancer recurrence, as well as the radiological data outlined above, the tissue will be collected and stored in a biobank as discussed in the consent section above. In the event of a recurrence, the data collected will be the same as a primary disease presentation.

Storage and analysis of biological samples

Tumour tissue biopsies at diagnosis, during treatment or on relapse will be obtained or at surgical resection at presentation, during treatment or on relapse in line with participant's consent.

The procedure for sample retrieval will depend on the nature of the sample requested and the laboratory services on site. If consent is withdrawn for issued samples by the donor, recipients will be informed of the relevant sample numbers and asked to return any unused samples for destruction. Results obtained from samples that have already been used for research need not be destroyed.

For peer review only - http://bmjopen.bmj.com/site/about/guidelines.xhtml

Routine imaging and radiomic analysis

All scans taken as part of routine care will be stored on hospital servers (as is usual practice). If not already in Newcastle, many of these scans will be transferred using the routine PACS transfer service, to Newcastle upon Tyne Hospital NHS Foundation Trust. These scans will be subjected to radiomic analysis: scans will be extracted in DICOM format anonymised ensuring removal of all patient identifiable information.

Statistical Analysis

Patient data and information will be collected, stored and used based upon patient consent, and consistent with GDPR requirements. Descriptive statistics will be used to describe the recruited cohort and data completion (at baseline and at follow-up). Baseline characteristics will be compared with routine data (from Hospital Episode Statistics) to determine whether the recruited sample is representative of laryngeal cancer patients across NHS in England.

Depending on the maturity of the cohort at the conclusion of the study, data will be used to start to develop risk prediction models and compare outcomes by treatment modality. Multiple logistic regression analyses will be used to (i) identify patient-related, clinical and health service-related (e.g. institution) factors associated with receipt of surgical vs non-surgical treatment; (ii) quantify locoregional control and identify factors associated with control overall and by treatment modality; and (iii) identify factors associated with quality-of-life overall and by treatment modality. Epidemiological approaches to support treatment comparisons within observational datasets (e.g. propensity scores, instrumental variable analysis) will be used. These analyses will provide an early demonstration of the value and potential of this new cohort.

The planned analyses will draw on a range of epidemiological analytical approaches. They will explore utility of high dimensional propensity scores (which incorporate additional variables in the propensity score, such as clinician and/or hospital characteristics, with these serving as proxies for unmeasured confounders) and instrumental variable analysis (which relies on the existence of an 'instrument', a variable that is related to the treatment but not to the study outcome other than through treatment effects).

Ethics and Dissemination

The study will be conducted in accordance with the recommendations for physicians involved in research on human subjects adopted by the 18th World Medical Assembly, Helsinki 1964 and later revisions. The majority of the interventions delivered take place as part of routine care, with LARCH providing a mechanism for recording this data. Biological material will be stored under each establishment Human Tissue Authority (HTA) licence. Ethical approval for the project has been obtained from London - Surrey Borders Research Ethics Committee, reference 22/PR/0406. The project has been included in the NIHR portfolio, CPMS ID: 52643. All potential future studies will have separate ethical approval.

Confidentiality and data management

The Caldicott Principles and General Data Protection Regulation (GDPR) will be fully adhered to when dealing with patient identifiable data. Within each recruiting site the Principal Investigator will preserve the confidentiality of participants taking part in the study. Within sites, no staff beyond the usual care team and local research team will have access to identifiable data. The data will be held at the site in

For peer review only - http://bmjopen.bmj.com/site/about/guidelines.xhtml

accordance with local Trust policies and will be destroyed following the study close in accordance with local Research and Development protocols.

The data controller (Sponsor) will have access to the full dataset but other users will be restricted to data from their own centre. Access will be via secure portal. Local sites will have access to their site data.

A purpose-built database and web portal will be developed to support the capture of patient recruitment and study participation data. Only authorised individuals will have login credentials to input this data. The staff (including research nurses within recruiting centres) will be able to remote access the portal to transfer the data. Staff will be a delegated out by Principal Investigator based on skills, training and experience; these will be used to restrict access to confidential or otherwise sensitive data. Our web portal will be deployed using REDCap is a browser-based Electronic Case Report Form (eCRF), suitable for Newcastle-led non-commercial research studies. This system site is on HSCN (i.e. the data is securely behind the NHS firewall).

Data generated by the research project will be retained after publications resulting from the research are finalised. Data will be stored for 20 years. After 20 years data will be considered for longer-term retention based on the published results and further advances in the field of research internationally. Datasets stored for >20 years will be anonymised.

At the end of the study, strategies to promote and publicise the cohort and processes for gaining access to data, samples or the recruitment network will be developed and widely publicized and promoted. These will include: information on the cohort website; designated contact point for further information or support; flyers, stalls or sessions at scientific and stakeholder conferences; webinars or podcasts, and providing a DOI created through data.ncl for inclusion on the project website and published papers.

The data sharing policy, and the procedures (including application forms) for requesting access to the cohort, will be made available on the cohort website. Currently it is anticipated that applications will be prioritised based on: quality; alignment with our vision; potential to translate into clinical practice or deliver significant patient or public benefit; collaborative approach; and added value. The cohort research committee will have responsibility for reviewing and approving/declining requests for access.

Data held in the cohort database will be offered for sharing once: (i) the principles on which applications will be reviewed, prioritised and approved are agreed within the collaboration; (ii) the processes for applying to access the cohort are agreed and implemented; (iii) reasonable numbers of patients have been recruited to the cohort. Investigators of adopted research studies will develop their own timelines for data sharing. A requirement of cohort approval will be that data are made available for sharing at as early a point as possible. For these studies, we currently anticipate that the applicant, research partner and other partners and collaborators will have access to the data for 1-2 years after the end of data collection for the study. Patients recruited to the cohort will be asked for consent to data sharing. For the cohort-adopted studies, it will be a requirement of adoption that consent is obtained from recruits for data sharing. The major delay to data sharing which will occur relates to the need to establish procedures for study review and adoption

The SMG will make the decision on data access. Committee membership will include the chief investigator (DWH) and co-investigators (JOH and LS) and PPI representation. This group will act as gatekeepers for the data. Decisions on whether or not an application is approved will be recorded

For peer review only - http://bmjopen.bmj.com/site/about/guidelines.xhtml

together with, in the event the application is declined, reasons and whether the applicants may re-apply if they fulfil certain conditions.

The Newcastle upon Tyne Hospitals NHS Foundation Trust, as data controller, will require data sharing agreements with all organisations responsible for submitting and accessing data.

Patients will have their date of birth and NHS number collected and this will be stored in a secure purpose built database. Where data linkage to national databases such as NHS Digital, National Cancer registries or Hospital Episode statistics is required, this identifiable information will be passed onto these national organisation to allow data linkage.

Patients who have withdrawn consent will have all data collected up until the point of withdrawal included in the study. This data will be uploaded onto an electronic CRF and included in the analysis of the study. Data will be submitted either directly onto the electronic CRF or onto paper CRF before input into the electronic CRF. In relation to consent for contact, researchers undertaking studies requiring access to identifiable data (e.g.in order to contact patients about participation in a new research study) will be granted appropriate access on the basis of agreement from the study management team and subject to ethical approval and consistent with patient consent.

Use of tumour samples or data by other researchers

Researchers may request to use material from the LARCH study which have been stored in Biobanks for future research. Researchers will be required to have their own NHS research ethics committee approval for their research projects, and the release of the material must be approved by the LARCH access committee to ensure an appropriate use of samples. This will be conducted in accordance with a formalised Access Policy and procedure. Access to the tissue collection is available to research groups based in the UK and elsewhere.

Patient and Public Involvement

We explored multiple potential study designs during the preparation of this protocol with three patient and public involvement (PPI) groups; these groups were not in favour of a randomised trial design. In defining precision medicine in laryngeal cancer, we do not aim to show whether a particular treatment is “better” for patients. Rather we aim to specify the tumour and patient factors which lead to a poor response to laryngeal cancer treatment (in general, and for specific treatments) in individual patients. PPI groups have checked and approved all patient facing study documents. We have a patient representative on the study management group and a patient (GS) is a co-author of this protocol and was involved in manuscript preparation

Sponsor

LARCH will be led by Newcastle University with The Newcastle upon Tyne Hospitals NHS Foundation Trust acting as sponsor. In participating sites, a principal investigator will be identified. Portfolio adoption will ensure that NIHR CRN research nurses are able to undertake patient recruitment.

Study management

LARCH will be coordinated by a study management group (SMG). The SMG will be chaired by the chief investigator and comprise co-investigators, PPI representation, study manager, research fellow, lead nurse, data managers and digital research engineers. The SMG will advise on matters including appropriateness and sensitivity of patient-facing materials; methods of patient approach, consent and data collection; strategies to maximise retention; interpretation of results; and dissemination to lay

For peer review only - http://bmjopen.bmj.com/site/about/guidelines.xhtml

audiences. Active involvement of the will help maximise patient acceptability and, hence, recruitment and retention. The study management team will govern all access to data.

A representative group of staff who will be part of the research team at each site will attend a site initiation meeting to ensure compliance with the protocol and allow training in study procedures and data collection methods. The PI at each study site will apply for local R&D approval. The PI will sign a copy of the ethically approved protocol to confirm agreement to carry out all study related tasks in accordance with the protocol. Deviations from protocol will be reported to the study management group.

Dissemination

Data from the study will be disseminated to participating centres within the study via the Trust's websites. Additionally, lay summaries will be prepared, posted on the study website and disseminated through websites of individual participating sites, charities, and locations advised by PPI. Digital tools for dissemination of research findings will be developed and information disseminated to individuals requesting this.

For academic and clinical dissemination, the results will be submitted for publication in high-impact international peer-reviewed journals and presented to scientific meetings.

Funding

Funding to this study is from The MRC/NIHR Clinical Academic Research Partnership (CARP; grant number MR/V037528/1), and is on NIHR portfolio (CPMS: 52643) gaining additional support in identifying patients and gaining consent.

Competing interests statement

All authors have no relationships with any companies that might have an interest in the submitted work in the previous 3 years and no non-financial interests that may be relevant to the submitted work.

Contributorship statement

All authors have made substantial contributions to the conception or design and drafting of the work. They have all approved of the version to be published and agree to be accountable for all aspects of the work. Authors' specific contributions are detailed as follows: DH: corresponding author, research question, research design, manuscript preparation, lead on revisions. JO'H: research question, research design, manuscript revisions. AR: research question, research design, manuscript revisions. GS: research design and conduct. MA: research governance, quality assurance, manuscript revision. KK: research governance and safety, manuscript revisions. RP: research governance and safety, manuscript revisions. DT: analysis strategy, manuscript revisions. JP: research design, outcome measures, manuscript revision. TMJ: research question, research design, manuscript revisions. LS: research question, research design, manuscript revisions

References

1. CRUK. Head and Neck Cancer Incidence Statistics 2015 [Available from: https://www.cancerresearchuk.org/health-professional/cancer-statistics/statistics-by-cancer-type/head-and-neck-cancers/incidence#heading-Four accessed 20/05/2019 2019.

For peer review only - http://bmjopen.bmj.com/site/about/guidelines.xhtml

2. Rachet B, Quinn MJ, Cooper N, et al. Survival from cancer of the larynx in England and Wales up to 2001. *British journal of cancer* 2008;99 Suppl 1:S35-7. doi: 10.1038/sj.bjc.6604581 [published Online First: 2008/10/01]

3. CRUK. Laryngeal cancer survival 2022 [Available from: https://www.cancerresearchuk.org/about-cancer/laryngeal-cancer/survival#:~:text=Generally%20for%20men%20with%20cancer,for%201%20year%20or%20more accessed 18th November 2022.

4. Hoffman HT, Porter K, Karnell LH, et al. Laryngeal cancer in the United States: changes in demographics, patterns of care, and survival. *The Laryngoscope* 2006;116(9 Pt 2 Suppl 111):1-13. doi: 10.1097/01.mlg.0000236095.97947.26

00005537-200609001-00001 [pii] [published Online First: 2006/09/02]

5. Pearce AM, Hanly P, Timmons A, et al. Productivity Losses Associated with Head and Neck Cancer Using the Human Capital and Friction Cost Approaches. *Appl Health Econ Health Policy* 2015;13(4):359-67. doi: 10.1007/s40258-015-0155-8 [published Online First: 2015/02/19]

6. Induction chemotherapy plus radiation compared with surgery plus radiation in patients with advanced laryngeal cancer. The Department of Veterans Affairs Laryngeal Cancer Study Group. *The New England journal of medicine* 1991;324(24):1685-90. doi: 10.1056/NEJM199106133242402

7. Forastiere AA, Goepfert H, Maor M, et al. Concurrent chemotherapy and radiotherapy for organ preservation in advanced laryngeal cancer. *The New England journal of medicine* 2003;349(22):2091-8. doi: 10.1056/NEJMoa031317

8. Hamilton DW, de Salis I, Donovan JL, et al. The recruitment of patients to trials in head and neck cancer: a qualitative study of the EaStER trial of treatments for early laryngeal cancer. *European archives of oto-rhino-laryngology : official journal of the European Federation of Oto-Rhino-Laryngological Societies* 2013 doi: 10.1007/s00405-013-2349-8

9. Urba S, Wolf G, Eisbruch A, et al. Single-cycle induction chemotherapy selects patients with advanced laryngeal cancer for combined chemoradiation: a new treatment paradigm. *Journal of clinical oncology : official journal of the American Society of Clinical Oncology* 2006;24(4):593-8. doi: 10.1200/JCO.2005.01.2047

10. Hamilton DW, Bins JE, McMeekin P, et al. Quantity vs quality of life in laryngeal cancer: a time trade off study. *Clinical Otolarygology* 2012;37(Suppl. 1):108.

11. DAHNO. National Head and Neck Cancer Audit 2014, DAHNO Tenth Annual Report 2014 [Available from: https://digital.nhs.uk/data-and-information/publications/statistical/national-head-and-neck-cancer-audit/national-head-and-neck-cancer-audit-2014-dahno-tenth-annual-report2019.

12. Ingarfield K, McMahon AD, Douglas CM, et al. Determinants of long-term survival in a population-based cohort study of patients with head and neck cancer from Scotland. *Head & neck* 2019 doi: 10.1002/hed.25630 [published Online First: 2019/01/09]

13. Birchall M, Bailey D, King P, et al. Effect of process standards on survival of patients with head and neck cancer in the south and west of England. *British journal of cancer* 2004;91(8):1477-81. doi: 10.1038/sj.bjc.6602118

14. NCRI. [Available from: https://www.ncri.org.uk/ncri-cancer-research-database-old/spend-by-research-category-and-disease-site/ accessed Oct 2020.

For peer review only - http://bmjopen.bmj.com/site/about/guidelines.xhtml

15. Economopoulou P, de Bree R, Kotsantis I, et al. Diagnostic Tumor Markers in Head and Neck Squamous Cell Carcinoma (HNSCC) in the Clinical Setting. *Front Oncol* 2019;9:827. doi: 10.3389/fonc.2019.00827 [published Online First: 2019/09/27]

16. Rajgor AD, Patel S, McCulloch D, et al. The application of radiomics in laryngeal cancer. *Br J Radiol* 2021;94(1128):20210499. doi: 10.1259/bjr.20210499 [published Online First: 2021/09/30]

For peer review only

STROBE Statement—checklist of items that should be included in reports of observational studies

	Item No	Recommendation	Page No
**Title and abstract**	1	(*a*) Indicate the study's design with a commonly used term in the title or the abstract	1,3
		(*b*) Provide in the abstract an informative and balanced summary of what was done and what was found	3
**Introduction**			
Background/rationale	2	Explain the scientific background and rationale for the investigation being reported	5,6
Objectives	3	State specific objectives, including any prespecified hypotheses	6
**Methods**			
Study design	4	Present key elements of study design early in the paper	7
Setting	5	Describe the setting, locations, and relevant dates, including periods of recruitment, exposure, follow-up, and data collection	9,10
Participants	6	(*a*) *Cohort study*—Give the eligibility criteria, and the sources and methods of selection of participants. Describe methods of follow-up<br>*Case-control study*—Give the eligibility criteria, and the sources and methods of case ascertainment and control selection. Give the rationale for the choice of cases and controls<br>*Cross-sectional study*—Give the eligibility criteria, and the sources and methods of selection of participants	7
		(*b*) *Cohort study*—For matched studies, give matching criteria and number of exposed and unexposed<br>*Case-control study*—For matched studies, give matching criteria and the number of controls per case	
Variables	7	Clearly define all outcomes, exposures, predictors, potential confounders, and effect modifiers. Give diagnostic criteria, if applicable	9,10
Data sources/ measurement	8*	For each variable of interest, give sources of data and details of methods of assessment (measurement). Describe comparability of assessment methods if there is more than one group	*9,10*
Bias	9	Describe any efforts to address potential sources of bias	11
Study size	10	Explain how the study size was arrived at	7
Quantitative variables	11	Explain how quantitative variables were handled in the analyses. If applicable, describe which groupings were chosen and why	11
Statistical methods	12	(*a*) Describe all statistical methods, including those used to control for confounding	11
		(*b*) Describe any methods used to examine subgroups and interactions	11
		(*c*) Explain how missing data were addressed	7,8
		(*d*) *Cohort study*—If applicable, explain how loss to follow-up was addressed<br>*Case-control study*—If applicable, explain how matching of cases and controls was addressed<br>*Cross-sectional study*—If applicable, describe analytical methods taking account of sampling strategy	7,8
		(*e*) Describe any sensitivity analyses	

Continued on next page

For peer review only - http://bmjopen.bmj.com/site/about/guidelines.xhtml

**Results**			
Participants	13*	(a) Report numbers of individuals at each stage of study—eg numbers potentially eligible, examined for eligibility, confirmed eligible, included in the study, completing follow-up, and analysed	N/A
		(b) Give reasons for non-participation at each stage	N/A
		(c) Consider use of a flow diagram	N/A
Descriptive data	14*	(a) Give characteristics of study participants (eg demographic, clinical, social) and information on exposures and potential confounders	N/A
		(b) Indicate number of participants with missing data for each variable of interest	N/A
		(c) *Cohort study*—Summarise follow-up time (eg, average and total amount)	N/A
Outcome data	15*	*Cohort study*—Report numbers of outcome events or summary measures over time	N/A
		Case-control study—Report numbers in each exposure category, or summary measures of exposure	N/A
		Cross-sectional study—Report numbers of outcome events or summary measures	N/A
Main results	16	(*a*) Give unadjusted estimates and, if applicable, confounder-adjusted estimates and their precision (eg, 95% confidence interval). Make clear which confounders were adjusted for and why they were included	N/A
		(*b*) Report category boundaries when continuous variables were categorized	N/A
		(*c*) If relevant, consider translating estimates of relative risk into absolute risk for a meaningful time period	N/A
Other analyses	17	Report other analyses done—eg analyses of subgroups and interactions, and sensitivity analyses	N/A
**Discussion**			
Key results	18	Summarise key results with reference to study objectives	N/A
Limitations	19	Discuss limitations of the study, taking into account sources of potential bias or imprecision. Discuss both direction and magnitude of any potential bias	N/A
Interpretation	20	Give a cautious overall interpretation of results considering objectives, limitations, multiplicity of analyses, results from similar studies, and other relevant evidence	N/A
Generalisability	21	Discuss the generalisability (external validity) of the study results	N/A
**Other information**			
Funding	22	Give the source of funding and the role of the funders for the present study and, if applicable, for the original study on which the present article is based	14

*Give information separately for cases and controls in case-control studies and, if applicable, for exposed and unexposed groups in cohort and cross-sectional studies.

Note: An Explanation and Elaboration article discusses each checklist item and gives methodological background and published examples of transparent reporting. The STROBE checklist is best used in conjunction with this article (freely available on the Web sites of PLoS Medicine at http://www.plosmedicine.org/, Annals of Internal Medicine at http://www.annals.org/, and Epidemiology at http://www.epidem.com/). Information on the STROBE Initiative is available at www.strobe-statement.org.

For peer review only
